# Exploring Causality between TV Viewing and Weight Change in Young and Middle-Aged Adults. The Cardiovascular Risk in Young Finns Study

**DOI:** 10.1371/journal.pone.0101860

**Published:** 2014-07-16

**Authors:** Harri Helajärvi, Tom Rosenström, Katja Pahkala, Mika Kähönen, Terho Lehtimäki, Olli J. Heinonen, Mervi Oikonen, Tuija Tammelin, Jorma S. A. Viikari, Olli T. Raitakari

**Affiliations:** 1 Paavo Nurmi Centre, Dpts of Physiology & Health and Physical Activity, University of Turku, Turku, Finland; 2 Unit of Personality, Work and Health Psychology, University of Helsinki, Helsinki, Finland; 3 Research Centre of Applied and Preventive Cardiovascular Medicine, University of Turku, Turku, Finland; 4 Department of Clinical Physiology, University of Tampere and Tampere University Hospital, Tampere, Finland; 5 Department of Clinical Chemistry, Fimlab Laboratories, Tampere University Hospital and School of Medicine, University of Tampere, Tampere, Finland; 6 LIKES Research Center for Sport and Health Sciences, Jyväskylä, Finland; 7 Department of Medicine, Turku University Hospital, Turku, Finland; 8 Department of Clinical Physiology and Nuclear Medicine, Turku University Hospital, Turku, Finland; Pennington Biomedical Research Center, United States of America

## Abstract

**Background:**

Television viewing time (TV time) is associated with increased weight and obesity, but it is unclear whether this relation is causal.

**Methods and Results:**

We evaluated changes in TV time, waist circumference (waist) and body mass index (BMI) in participants of the population-based Cardiovascular Risk in Young Finns study (761 women, 626 men aged 33–50 years in 2011). Waist and BMI were measured, and TV time was self-reported in 2001, 2007, and 2011. Changes in waist and BMI between 2001 and 2011 were studied a) for the whole group, b) in groups with constantly low (≤1 h/d), moderate (1–3 h/d), or high (≥3 h/d) TV time, and c) in groups with ≥1 hour in-/decrease in daily TV time between 2001 and 2011. BMIs in 1986 were also evaluated. We explored the causal relationship of TV time with waist and BMI by classical temporality criterion and recently introduced causal-discovery algorithms (pairwise causality measures). Both methods supported the hypothesis that TV time is causative to weight gain, and no evidence was found for reverse or bidirectional causality. Constantly low TV time was associated with less pronounced increase in waist and BMI, and waist and BMI increase was lower with decreased TV time (P<0.05). The increase in waist and BMI was at least 2-fold in the high TV time group compared to the low TV time group (P<0.05). Adjustment for age, sex, BMI/waist in 2001, physical activity, energy intake, or smoking did not change the results.

**Conclusions:**

In young and middle-aged adults, constantly high TV time is temporally antecedent to BMI and waist increase.

## Introduction

Sedentary time is defined as physically inactive time passed mostly sitting, during which energy consumption is close to the resting state (1–1.5 MET). Sedentary behavior, especially TV viewing (TV time), is associated with obesity [1], and cardio-metabolic disorders, such as metabolic syndrome [2], type 2 diabetes, cardiovascular diseases [1], [3]–[8], and premature death [6], [9]–[12]. Obesity is known to increase the risk of cardio-metabolic disorders, and it may be an important link between TV time and cardio-metabolic outcomes. Therefore, TV time could be a modifiable behavioral factor with potential effects in obesity prevention. At present, however, it is not known whether TV time causes weight increase, or whether weight increase leads to sedentariness and increased TV time. Evidence supporting both scenarios have been reported. Most longitudinal studies have found consistent relations between TV time and weight gain from childhood to the adult years [13]. However, findings have been mixed for associations with weight gain during adulthood [13]. In some longitudinal studies, prior obesity [14] or increased fat mass [15] have been identified as risk factors for increased TV time. In addition, we have previously observed a direct association between a genetic obesity risk score for high BMI and sedentary time in men [16]. These findings suggest that also high body weight may be causally related to TV time. Nevertheless, because TV viewing decreases energy expenditure and possibly increases energy intake [17]–[19], it is generally hypothesized that TV time causes weight increase. Therefore, intervention studies have been initiated with the attempt to induce weight loss by reducing TV time. Most intervention studies, but not all [20]–[24], have observed weight reduction, but it may vary depending e.g. on the age of the target group. In one randomized controlled trial, TV viewing time was reduced by 50% in 36 overweight and obese adults over a 3-week period, and this study showed a significant increase in objectively measured energy expenditure resulting in decreased energy intake and BMI [20]. Another intervention study in children showed that a 50% reduction in TV and computer use produced significant reductions in BMI and energy intake, but no changes in physical activity [21]. A third intervention study in 192 children [22] demonstrated that a reduction of TV time was associated with weight reduction.

Making etiogenetic causal inferences from associations has its well-known limits. However, the use of longitudinal data and novel statistical methods may provide opportunities to test causal hypotheses. Causal relationship can be best studied in a randomized clinical trial setting, but long-term clinical trials to study the effects of interventions in larger populations (e.g. reduction of sedentary time) are practically almost impossible to conduct. Longitudinal datasets with repeated measurements offer a good possibility to test the direction of the causality with the classical temporality criterion [25], and such analysis can be complemented with recently introduced causality-estimation algorithms that take advantage of higher moments of distributions to allow exploration of causal inferences [26]–[29]. This additional perspective is valuable as Hill originally noted upon introducing his causality criteria that “None of my nine viewpoints can bring indisputable evidence for or against the cause-and-effect hypothesis and none can be required as sine qua non. What they can do, with greater or less strength, is to help us make up our minds on the fundamental question – is there any other way of explaining the set of facts before us, is there any other answer equally, or more, likely than cause and effect” [25].

The aim of our study was to explore the relative importance of the two previously suggested causal directions: that from TV time to obesity and the opposite one from obesity and associated physical restrictions to TV time. We took a public-health perspective, aiming to detect the dominant causality in the general population, acknowledging that individuals displaying both pathways are likely to exist.

We analyzed the development of waist circumference and BMI during 10 years of follow-up in subjects with different amounts of daily TV viewing time. The BMIs 14 years prior to this period were also evaluated. In addition to assessing temporal relations, we utilized two different causality-estimation algorithms to explore whether TV time was causative for waist and BMI change.

## Methods

### Ethics statement

The participants gave a written informed consent, and the study was approved by local ethics committees (The Ethics Committee of the Hospital District of Southwest Finland).

### Participants

The Cardiovascular Risk in Young Finns Study is an ongoing, multicenter follow-up study of atherosclerosis risk factors [30]. The first cross-sectional survey was conducted in 1980, when 3,596 individuals aged 3–18 years participated. These participants were randomly chosen from the national registry of the study district. Since 1980, several follow-up studies have been conducted. The latest 30-year follow-up survey was performed in 2011 when 2,060 of the original participants (aged 33–50 years) attended. The participants gave a written informed consent, and the study has been approved by local ethics committees.

### Assessing TV viewing time

A self-administered questionnaire was used to collect data on daily TV viewing time (TV time). TV time was the measure of sedentary behavior in this study, since among the various non-occupational sedentary behaviors in this population, and also most frequently in other studies, TV time has been associated with weight increase and various health risks [16].

The participants were asked how much time on average they spent watching TV daily. In 2001 and 2011 the daily TV time was recorded in minutes, and in 2007 in one-hour increments (from 0 to 9 hours or ≥10 hours). In 2011, weekday and weekend TV times were recorded separately. TV hours in 2007 were transformed into minutes, and a mean daily TV time in 2011 was calculated.

The study population was divided in groups with different TV times, i.e. constantly “low” (≤1 h, n = 200), “moderate” (1–3 h, n = 238), or “high” (≥3 h, n = 84) daily TV time in 2001, 2007, and 2011. In addition “increased” (n = 221) and “decreased” (n = 216) groups reporting at least a 1-hour increase or decrease in their daily TV viewing time between 2001 and 2011 were created. The cut-off points were selected to provide practically useful time categories. 428 study participants did not fulfill these TV time group criteria.

### Body mass index and waist circumference

Weight was measured with a digital scale in light clothing without shoes with an accuracy of 0.1 kg, and height with a wall-mounted stadiometer with an accuracy of 0.1 cm. BMI was calculated as weight (kg)/[height (m)]^2^. Waist circumference was measured with an anthropometric tape in the end of expiration at the mid-axillary line between the iliac crest and the lowest rib with an accuracy of 0.1 cm. BMI measured in 1986 (at ages 9–24) was selected to represent the prior BMI.

### Physical activity, energy intake, smoking

Physical Activity Index in 2001, 2007, and 2011 was calculated based on self-reported leisure-time physical activity, its frequency, duration, and intensity.

Energy intake in 2007 was assessed using a 131-item food frequency questionnaire (FFQ), developed and validated by the Finnish National Institute for Health and Welfare [31].

Smoking habits were collected in 2001, 2007, and 2011 with a self-administered questionnaire. Individuals who reported smoking daily were considered as smokers.

### Statistical Analyses

#### Study setting

All 1,387 participants (761 women, 626 men) with complete data on BMI, waist and daily TV time at 2001, 2007, and 2011 were included in this study.

#### Longitudinal analyses of the BMI and waist circumference change

The 10-year changes in waist and BMI from 2001 to 2011 were evaluated for the whole study population, and for the different TV time groups. Mean waist and BMI at each follow-up, and the changes in waist or BMI between 2001 & 2007, and 2001 & 2011 were calculated. The BMIs from 1986 were used to assess the mean BMI in different TV time groups prior to the baseline of 2001. Waist data prior to 2001 were not available.

As TV viewing time was associated with waist and BMI, both in males and females, and the only sex-by-TV interaction was seen in 2011 with BMI as outcome (p<0.02), the longitudinal analyses were performed with sexes combined. Sex differences for age, and TV time within each group were analyzed with non-parametric Wilcoxon 2-sample test. The associations of TV time with waist and BMI at each time point in each group were studied with linear regression. Waist and BMI differences in TV time groups were studied with linear regression, multiple comparison corrected (Tukey-Kramer) test. In addition, the risk ratios (RRs) for obesity defined by BMI>30 were calculated using generalized linear modelling. All these analyses were adjusted by sex, age, mean Physical Activity Index and smoking in 2001, 2007 and 2011, and energy intake in 2007. The statistical analyses for longitudinal change in waist and BMI were done with the SAS version 9.2, and statistical significance was inferred at a 2-tailed probability value <0.05.

#### Exploring causality

Bradford Hill provided in his classic paper on causation a list of additional aspects that one should especially consider for an observed association before deciding on the most likely interpretation of its causation [25]. One of these criteria is temporality. Hill's example is analogous to our question whether abundant TV watching leads to obesity in the long run or obesity to spending a lot of time in front of TV. We define temporality by achieved level of variable A predicting future change of variable B. When this relationship is found for achieved levels of A only, and not clearly for achieved levels of B predicting future change of A, temporality criterion speaks for the causal antecedence of A. In addition to the temporality criterion, we define another, more recent criterion for causality, and use it for incremental validity, as Hill noted that “None of my nine viewpoints can bring indisputable evidence for or against the cause-and-effect hypothesis and non can be required as sine qua non. What they can do, with greater or less strength, is to help us make up our minds on the fundamental question – is there any other way of explaining the set of facts before us, is there any other answer equally, or more likely than cause and effect”.

We studied whether TV time was causative for waist and/or BMI change utilizing two different methods. First, we evaluated whether the baseline value of the antecedent variable was more strongly associated with subsequent progression of the descendent variable, or *vice versa*, according to classical temporality criterion [25]. Secondly, we applied the recently introduced distribution-based pairwise causality estimates, where the direction of causality can be determined even from cross-sectional data. The pairwise causality estimation, as applied here, starts from the assumptions that (a) either obesity, x_o_, causes TV time or TV time, x_t_, causes obesity, (b) the causal association is linear, (c) independent residual terms are non-Gaussian (distributed according to some other than the Normal distribution), and (d) there are no (strongly/fully) confounding variables. This is a Linear, Non-Gaussian, Acyclic Model {LiNGAM [26]}. Mathematically it means that for centered (zero-mean) variables either
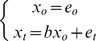
(1)or
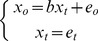
(2)


holds, where e_o_ and/or e_t_ is a non-Gaussian variable, and b is a constant, non-zero regression coefficient. The aim of the causality algorithms is to estimate which one holds, the system of equations 1 or the system of equations 2. In these two alternative systems of equations, either obesity or TV time is an exogenous variable: an exogenous variable is not predicted by other variables in the system, and can be considered as an input to a system of variables. The estimated exogenous variable is causal, because the other variable is its function, and it is not a function of the other variable. In other words, manipulations of an exogenous variable lead to changes in the other (endogenous) variable, but manipulations of an endogenous variable do not affect the exogenous variable.

With non-Gaussian variables and the LiNGAM model, one may determine causality by estimating which one is the exogenous variable, x_o_ or x_t_, by estimating which one is less dependent on its residuals [27]. In the DirectLiNGAM-algorithm [27], this dependency is evaluated using a nonparametric, kernel-based estimator [32] of the mutual information between two variables [33]. In addition, other pairwise measures can be constructed [29]. Despite the measure, this general strategy does not work for Normal distributions, because they are fully described by their means and covariances, and covariance between a regression residual and corresponding independent variable is always zero by definition. For Gaussian variables then also statistical dependency and mutual information is zero, whereas non-Gaussian variables contain additional information (skewness, kurtosis, etc.) to be used. Two different pairwise measures of causality, DirectLiNGAM-based and entropy-based [27], [29], were applied here. For each statistic, a positive value signifies causal antecedence of the first argument/variable, and a negative value indicates the opposite condition.

If one denotes by M(x_o_,x_t_) the mutual information between x_o_ and ordinary least squares regression-residual of x_t_ (estimating e_t_ in Eq. 1), and by M(x_t_,x_o_) the mutual information between the opposite configuration, then under the LiNGAM assumptions the inequality M(x_o_,x_t_)<M(x_t_,x_o_) implies that x_o_ is the causal antecedent and vice versa [27]. Therefore, one can use the quantity
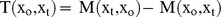
as a causality statistic, the positive values of which indicate that x_o_ causes x_t_, whereas the negative values indicate the opposite. When applying the exact same kernel-based pairwise quantity M(·,·) that the DirectLiNGAM-algorithm uses [27], we refer to this statistic T as the kernel-based statistic T_kernel_. As an additional sensitivity analysis, we provide results from Hyvärinen's and Smith's [29] entropy-based approximation of M(·,·), referring to ensuing statistic as T_entropy_. More restricted deviations from Gaussianity could also be used for the causality estimation in special cases. Particularly conceptually illuminating is the case of skewed variables.

Although use of skewness-based statistic is not recommended for general cases, we describe it following Hyvärinen and Smith [29] to give the reader a concrete intuition on why information in third moments can allow causal inference in LiNGAM. Let variables x_o_ and x_t_ be standardized (mean zero, variance one) variables with positive skewness, E the expectation operator, and r(x_o_,x_t_) the correlation between x_o_ and x_t_. Then the desired skewness-based statistics is
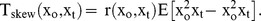



The sign-requirement is not a limitation, as if a variable x* has a negative skewness, then the statistics can nonetheless be applied to x =  sign(skew(x*))x*; that is, a skewed variable multiplied by the sign of its skewness always has a positive skew. The statistic can be understood as follows:

If x and y are standardized variables with positive skewnesses and y = rx + e holds, we have T_skew_(x,y) = r(E[x^3^r+e] - E[x(rx+e)(rx+e)]). Using standard calculus for expectations, independence of x from error e, and the fact that E[x^3^] =  skew(x) for a standardized variable x, one easily obtains that T_skew_(x,y) =  skew(x)(r^2^–r^3^). As skew(x) >0, and |r|<1, it follows that T_skew_(x,y)>0. But when x = ry+e holds, similar calculations yield T_skew_(x,y) =  skew(y)(r^3^–r^2^)<0. Hence, if x is cause under the linear model, this is detected by the positive values of the statistic T_skew_(x,y), and the causality from y to x is detected by the negative values. This proves that causality can sometimes be inferred from cross-sectional observations, under specific constraints. Derivations of the general measures rely on more complex information-theoretic arguments, but the basic idea is similar.

Despite rather strict assumption in principle, we have previously shown by simulation that, in practice, partial confounding is well-tolerated by the Kernel-based algorithm [28]. The ability to detect the causal antecedent decreased smoothly as a function of the degree of confounding until both variables were fully caused by a third variable and had no direct causal link, when the algorithm was indecisive (i.e., both variables were causal in ∼50% of bootstrap replications). The methods are not sensitive to measurement errors either. The 95% bootstrap-percentile confidence intervals for causality statistics were derived from 2000 bootstrap resamples [34]. Missing-data imputation methods are not available for pairwise causality statistics, and therefore bootstrap resamples were drawn from full data and pairwise non-complete observations dropped per individual resample and comparison.

**Figure 1:**
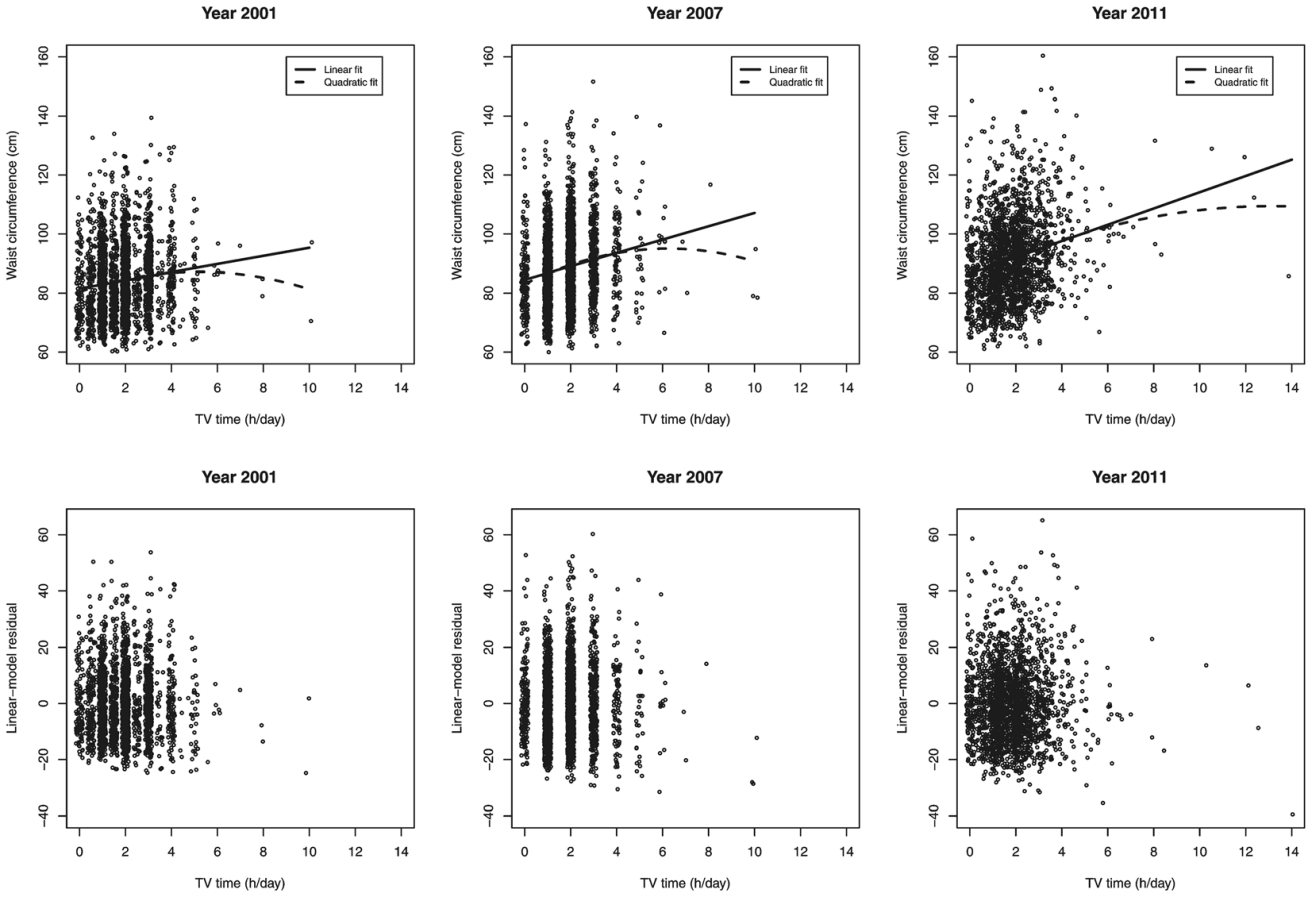
Linear Regression Models with a quadratic term (dashed line) and without it (solid line). Residual plots are for the model with only the linear term included. Jitter, that is a uniform random variable on the interval [−0.3, 0.3], was added to x-axis for enhanced discernibility, but did not enter to model estimation.

The assumptions of non-Gaussian distribution for the pairwise causality estimates were tested using standard Kolmogorov-Smirnov tests for deviations from normality; these were significant for all studied variables (each p<.001), as required. To provide further qualitative information on the deviations from Gaussian distribution, D'Agostino's tests for skewness and Anscombe-Glynn test for kurtosis are reported along other basic statistics (Table 1). Next, standard linear regression models were estimated, and the independence between residuals and the independent variable was evaluated using the non-parametric Hoeffding's test. Figure 1 shows the linear-model fits when predicting waist with TV time. A clear linear effect was observed (*e.g.* adjusted *R^2^* = 0.015 in 2001 follow-up), as well as a small quadratic effect (*P* = 0.011, adjusted *ΔR^2^* = 0.002, in 2001). The non-parametric Hoeffding's test did not reject the assumption of independence between TV time and linear-regression residual of waist circumference required for causality estimation (*P* = 0.225 in 2001; *P* = 0.073 in 2007; and *P* = 0.439 in 2011). Hence, the required assumptions for pairwise causality estimation for waist and TV time were adequately fulfilled. Similar results were obtained for BMI (not shown).

**Table 1:** Basic Characteristics for temporal sequence analyses.

Variable (unit)	Mean	SD	Skewness	Kurtosis	n
Waist (cm)					
In 2001	84.1	12.3	0.74	3.59	2253
In 2007	88.6	13.5	0.71	3.78	2181
In 2011	91.9	14.2	0.72	3.98	2051
**BMI (kg/m^2^)**					
In 2001	25.1	4.4	1.15	5.30	2276
In 2007	26.0	4.8	1.27	6.29	2170
In 2011	26.5	5.1	1.26	5.87	2049
**TV time (h/day)**					
In 2001	1.9	1.2	1.05	6.01	2594
In 2007	1.8	1.1	1.40	8.47	2224
In 2011	1.9	1.2	2.02	15.45	1970

All skewnesses and kurtoses are statistically significantly different from those of the Normal distribution (skewness  = 0, kurtosis  = 3), at the 0.001 significance level, and therefore non-Gaussian as required by the pairwise causality estimates.

Waist  =  Waist circumference.

BMI =  Body mass index.

SD =  Standard deviation.

n =  All available observations for the variable in question.

The statistical causality analyses were performed using Matlab-software version R2012a 7.14.0 with the previously provided additional codes [28], [29], and their assumptions tested with R-software version 2.15.2 [35], with “Harrell miscellaneous” (cran.r-project.org/web/packages/Hmisc/), and “moments” packages (cran.r-project.org/web/packages/moments/).

The 959 participants distributed to five separate TV time groups for the purpose of the longitudinal BMI/waist change analyses left a statistical power of 0.956 for rejecting null hypothesis of no difference given that a small effect (f∧2 = 0.02) actually exists and significance level of 0.05 is used. Medium and large group differences can be detected with certainty (power  = 1). Regarding the causality methods, we have performed our previous simulations in approximately similarly sized random samples, showing a reasonable power [28]. Herein, we give an example of power calculation for the present context in the Text S1.

All relevant codes for conducting the cross-sectional pairwise causality estimation and related bootstrap estimates of uncertainty have previously been made available [27]–[29], and the other data used herein is available for re-examination from the corresponding author upon request.

## Results

### Characteristics

The mean ages and TV times for males and females in different TV time groups were similar (p>0.05, Table 2).

**Table 2:** Age in 2011, and TV time at various time points by TV time group (constantly low, moderate, high, increased, or decreased) and by sex.

	SEXES COMBINED			FEMALES			MALES			Sex difference
ALL	n		SD	n		SD	n		SD	p-value
**Age in 2011** (y)	1387	42.2	5.0	761	42.2	5.0	626	42.1	5.0	0.84
**TV time** (min/day)										
**In 2001**	1387	109.0	66.1	761	103.2	63.2	626	116.2	68.9	0.0002
**In 2007**	1387	105.2	63.6	761	102.7	63.6	626	108.4	63.5	0.069
**In 2011**	1387	111.1	68.6	761	106.1	63.2	626	117.2	74.3	0.011
**CONSTANTLY LOW TV TIME** (≤1 h/day in 2001, 2007 and 2011)										
**Age in 2011** (y)	200	42.1	4.8	115	41.7	4.8	85	42.7	4.9	0.14
**TV time** (min/day)										
**In 2001**	200	34.4	24.0	115	34.0	23.0	85	34.9	25.4	0.70
**In 2007**	200	36.6	29.3	115	36.0	29.5	85	37.4	29.2	0.74
**In 2011**	200	31.8	22.9	115	31.4	22.9	85	32.4	23.1	0.70
**CONSTANTLY MODERATE** **TV TIME** (1–3 h/day in 2001, 2007 and 2011)										
**Age in 2011** (y)	238	42.5	5.0	138	42.4	5.1	100	42.6	5.0	0.87
**TV time** (min/day)										
**In 2001**	238	115.5	15.9	138	114.8	17.4	100	116.4	13.7	0.36
**In 2007**	238	120.0	0	138	120.0	0	100	120.0	0	1.0
**In 2011**	238	119.9	27.0	138	121.9	27.4	100	117.1	26.3	0.22
**CONSTANTLY HIGH TV TIME** (≥3 h/day in 2001, 2007 and 2011)										
**Age in 2011** (y)	84	42.0	5.1	36	41.3	5.2	48	42.6	5.0	0.28
**TV time** (min/day)										
**In 2001**	84	216.4	57.8	36	218.3	39.7	48	215.0	68.7	0.19
**In 2007**	84	221.4	72.7	36	231.7	92.0	48	213.8	53.8	0.60
**In 2011**	84	228.2	55.5	36	225.8	50.2	48	229.9	59.7	0.8
**INCREASED TV TIME** (increased with ≥1 h/day between 2001 and 2011)										
**Age in 2011** (y)	221	43.7	4.9	111	44.0	4.9	110	43.3	4.9	0.25
**TV time** (min/day)										
**In 2001**	221	72.5	43.3	111	68.1	42.8	110	76.9	43.4	0.21
**In 2007**	221	110.5	55.4	111	108.6	61.9	110	112.4	48.2	0.63
**In 2011**	221	171.6	74.6	111	164.4	61.1	110	178.8	85.8	0.34
**DECREASED TV TIME** (decreased with ≥1 h/day between 2001 and 2011)										
**Age in 2011** (y)	216	40.2	4.6	107	40.1	4.5	109	40.3	4.6	0.67
**TV time** (min/day)										
**In 2001**	216	176.4	62.6	107	172.4	64.8	109	180.4	60.5	0.25
**In 2007**	216	112.8	62.2	107	111.0	54.5	109	114.5	69.1	0.94
**In 2011**	216	74.1	48.3	107	70.6	51.8	109	77.5	44.5	0.16

SD =  Standard deviation.

n =  All available observations for the variable in question.

The distribution of BMI measured in 1986 was similar between the groups with constantly low, moderate or high TV time (Table 3). The group that increased TV time between 2001 and 2011 had 9% higher BMI in 1986 than the group that decreased TV time during the same time period. In 2001, the constantly high TV time group had a 5% larger waist, and 7% higher BMI than the constantly low TV time group. BMI measured in 2001 was 5% higher in the constantly moderate TV time group compared to the constantly low TV time group (P<0.05 in all, Table 3).

**Table 3:** Mean waist circumference and BMI in 1986, 2001 and 2011, and change in waist/BMI between 2001 & 2007, and 2001 & 2011 in different TV time groups, with Tukey-Kramer corrected pairwise TV group comparisons.

TV time between 2001 and 2011 (n)	1986	2001		2011		Change from 2001 to 2007*		Change from 2001 to 2011*	
	BMI (kg/m^2^)	Waist (cm)	BMI (kg/m^2^)	Waist (cm)	BMI (kg/m^2^)	Waist (cm)	BMI (kg/m^2^)	Waist (cm)	BMI (kg/m^2^)
**Constantly low** (200)	20.2	82.7	24.3	89.4	25.9	3.0	0.5	5.0	0.8
**Constantly moderate** (238)	20.4	85.4	25.4^1^	92.5^1^	26.7^1^	5.5^1^	1.2^1^	8.4^1^	1.7^1^
**Constantly high** (84)	20.6	86.9^1^	26.0^1^	94.9^1^	27.5^1^	6.7^1^	1.7^1^	10.9^1^	2.5^1^
**Increased** (221)	21.2	85.0	25.2	92.7^1^	26.9^1^	5.0^1^	1.2^1^	8.3^1^	1.8^1^
**Decreased** (216)	19.4^2^^,^ 4	84.0	25.0	91.7^1^^,^ 3	26.4^3^	4.8	0.9^3^	7.4^3^ ^1^^,^ 3	1.3^3^
**All** (1387)	20.3	83.9	24.9	91.6	26.5	4.6	1.0	7.7	1.6

n =  All available observations for the variable in question.

Waist  =  Waist circumference.

BMI =  Body mass index.

Constantly low  =  TV time ≤1 h/day in 2001, 2007 and 2011.

Constantly moderate  =  TV time >1 h, but <3 h/day in 2001, 2007 and 2011.

Constantly high  =  TV time ≥3 h/day in 2001, 2007 and 2011.

Increased  =  TV time increased with ≥1 h/day between 2001 and 2011.

Decreased  =  TV time decreased with ≥1 h/day between 2001 and 2011.

* =  adjusted by sex, age, physical activity, energy intake, smoking, and Waist or BMI in 2001.

Tukey-Kramer adjusted pairwise comparisons:

1 = statistically significant difference with Low group (p<0.05).

2 = statistically significant difference with Moderate group (p<0.05).

3 = statistically significant difference with High group (p<0.05).

4 = statistically significant difference with Increase group (p<0.05).

Most of the TV time change (≥1 hour) in the increase and decrease groups occurred on a moderate TV time level (1–3 h/day; Table 2). The mean TV time in the group that increased TV time was 72 min/day in 2001 and 172 min/day in 2011 (an increase of 139%). The mean TV time in the group that decreased TV time was 176 min/day in 2001 and 74 min/day in 2011 (a decrease of 58%).

### Longitudinal change in waist circumference and BMI

**Figure 2:**
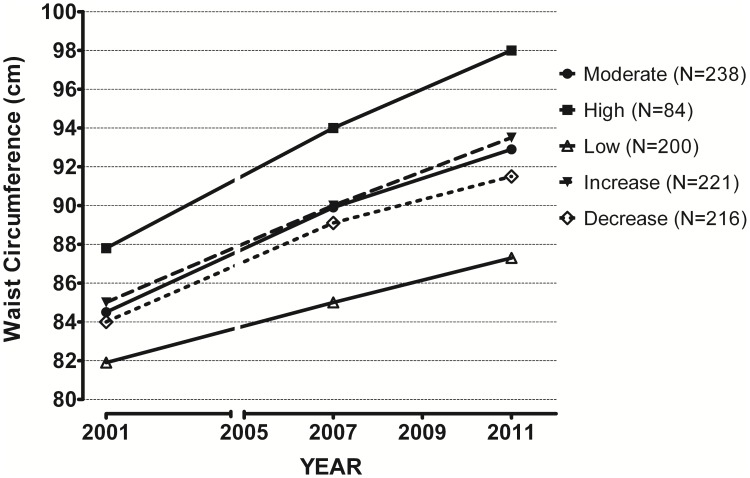
Waist circumference change during 10 years of follow-up depending on daily TV time, and its stability or change.

**Figure 3:**
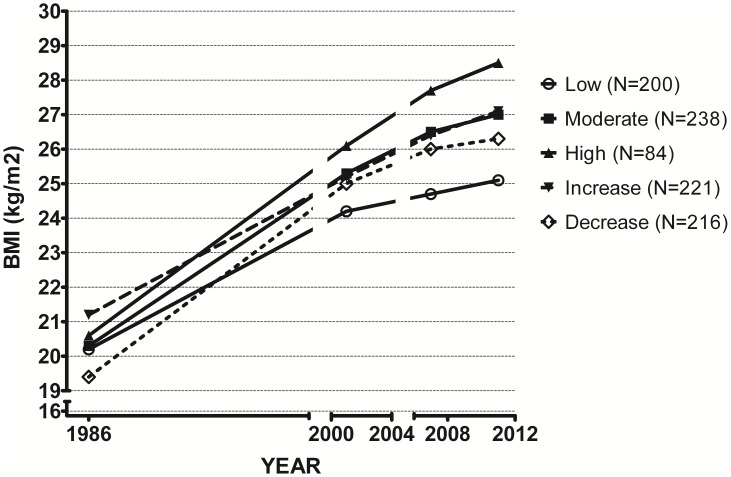
BMI change during 10 years of follow-up depending on daily TV time, and its stability or change. In addition, the BMI from 1986 (14 years prior) is shown.

Overall, in comparison to the constantly low TV time group, the waist and BMI increased more in the constantly moderate and constantly high TV time groups, but also in those that increased their TV time with 1 h/day during the 10-year period (p<0.05 in all, Table 3, Fig 2–3). At the same time when compared to the group with constantly high TV time, waist and BMI increased less in the group that decreased their TV time (p<0.05). Increase in waist and BMI during the 10-year period was approximately 2-fold in the group with constantly high TV time compared to the increase seen in the group with constantly low TV time (Table 3). Adjustments for sex, age, baseline BMI/WC, physical activity, energy intake and smoking did not change the results. In addition, the risk ratios for obesity calculated in different TV time groups using generalized linear modelling showed an increased risk with increased TV viewing time (Table S1).

### Causality explorations

TV time correlated positively both with waist and BMI in 2001, 2007, and 2011 (Pearson's r≥0.078 in all; Table 4). Furthermore, TV time in 2001 predicted subsequent increase of both BMI and waist, but neither BMI nor waist at baseline predicted changes in TV time. Also the kernel-based measure of causality indicated that TV time was causally antecedent for BMI and waist increase, although the measure did not reach statistical significance in all pairwise comparisons (Table 4). No suggestion of a reverse causal relationship was seen in the pairwise analyses.

**Table 4:** Correlations and Pairwise Causality Statistics between the Study Variables.

	Comparison	r (95% CI)	T_kernel_ (95% CI)	T_entropy_ (95% CI)
**Cross-sectional**	TV-time vs. Waist, 2001	**0.126 (0.085, 0.167)**	**0.015 (0.000, 0.032)**	0.001 (−0.006, 0.007)
	TV-time vs. Waist, 2007	**0.187 (0.146, 0.227)**	**0.175 (0.097, 0.276)**	−0.011 (−0.027, 0.001)
	TV-time vs. Waist, 2011	**0.203 (0.160, 0.245)**	0.011 (−0.009, 0.037)	−0.014 (−0.052, 0.022)
	TV-time vs. BMI, 2001	**0.116 (0.075, 0.156)**	0.014 (−0.006, 0.030)	−0.001 (−0.008, 0.006)
	TV-time vs. BMI, 2007	**0.185 (0.144, 0.225)**	**0.164 (0.091, 0.260)**	−0.012 (−0.029, 0.003)
	TV-time vs. BMI, 2011	**0.170 (0.127, 0.213)**	0.005 (−0.018, 0.031)	−0.020 (−0.0512, 0.005)
				
**Longitudinal**	TV-time vs. Δ_6y_Waist	**0.101 (0.055, 0.148)**	**0.018 (0.004, 0.039)**	−0.001 (−0.007, 0.006)
	Waist vs. Δ_6y_TV-time	0.011 (−0.035, 0.057)	−0.001 (−0.014, 0.001)	0.000 (−0.002, 0.002)
	TV-time vs. Δ_10y_ Waist	**0.110 (0.062, 0.157)**	**0.023 (0.008, 0.043)**	0.000 (−0.005, 0.008)
	Waist vs. Δ_10y_TV-time	0.030 (−0.019, 0.078)	−0.001 (−0.011, 0.001)	−0.002 (−0.017, 0.002)
	TV-time vs. Δ_6y_BMI	**0.078 (0.032, 0.124)**	**0.012 (0.001, 0.030)**	0.000 (−0.007, 0.007)
	BMI vs. Δ_6y_TV-time	0.020 (−0.026, 0.066)	−0.001 (−0.017, 0.001)	0.000 (−0.002, 0.003)
	TV-time vs. Δ_10y_BMI	**0.085 (0.038, 0.133)**	**0.014 (0.001, 0.035)**	0.001 (−0.005, 0.009)
	BMI vs. Δ_10y_TV-time	0.018 (−0.031, 0.066)	0.000 (−0.006, 0.002)	0.000 (−0.011, 0.003)

Positive value of *T_kernel_* or *T_entropy_* suggests that the first-mentioned variable in comparison is causal antecedent of the secondly mentioned, whereas a negative value implies the opposite. Parentheses give 95% bootstrap-percentile confidence intervals of estimates, except for ordinary correlation for which standard asymptotic theory was used. Statistically significant comparisons are highlighted with bold font.

Δ_6y_ =  change over six years (from 2001 to 2007).

Δ_10y_ =  change over ten years (from 2001 to 2011).

Waist  =  Waist circumference.

*r* =  Correlation coefficient.

*T_kernel_* =  DirectLiNGAM- and Kernel-based measure of pairwise causality.

*T_entropy_* =  Approximate-entropy and asymptotic-likelihood –based measure of pairwise causality.

## Discussion

In this population-based longitudinal study in young and middle-aged adults, constantly high TV time during 10-year period was associated with larger increases in waist and BMI. The increases were on average 2-fold in the group with constantly high TV time when compared to the group with constantly low TV time. Both the classical temporality criterion and novel pairwise causal-discovery algorithm suggested that TV time is causally antecedent to BMI and waist increase. We found no evidence for reverse or bidirectional causality suggested in some previous studies [14], [15]. These data add to the increasing body of evidence on the health risks related to sedentary lifestyle.

The mechanism behind the obesogenic effect of TV viewing is still unclear, but according to our previous cross-sectional (16) and other interventional studies [21], [22] it may be partially mediated by other clustered, unhealthy behaviors, e.g. diet, and other risks for obesity. Prolonged TV viewing may also displace physical activity [11], as seen when TV time is experimentally reduced [20]. There is evidence on the harmful effect of prolonged sitting on skeletal muscle gene expression [36], but the health risks associated with sedentary behavior may also be mediated by increase in weight. Prolonged and abundant sitting may cause increased cardio-metabolic disease risk also through other, still unknown, direct mechanisms.

A limitation of this study is that TV time, physical activity and diet were collected using questionnaires, and that the measures changed slightly between follow-ups. When compared to the national TV viewing time statistics in Finland [37], the daily TV times reported in this study were below the mean national level, indicating that the reported time may more likely be an underestimate. In general, data collected with questionnaires may be associated with recall bias of e.g. physical activity/inactivity, diet, etc, and they may at times result in (un)intentional over- or underestimation of the collected data, but they are most probably accurate enough in distinguishing the magnitudes and trends in a larger population. TV viewing time used in our study as a measure of sedentary behavior is a more concrete and simple measure that may be recalled more accurately than e.g. overall sedentary time, and is therefore most probably adequately reliable even if self-reported.

On the other hand, current objective measures cannot well distinguish TV viewing from other inactivity. Neither do they easily distinguish sedentary time from low intensity physical activity - especially, if HR monitors and their data are used like they did in the Ekelund study [15]. Mixing low intensity physical activity with sedentary time would easily dilute the results of any sedentary time analysis. Current objective measurements are also incapable of distinguishing various forms of sedentary behaviors supporting the use of some kind of questionnaires. One must also remember that monitoring devices may turn to be less objective than expected, since they may modify one's behavior, however closely their use and behavior during the use is guided.

A change in TV time in this study was reflected in waist and BMI, but as most of the ≥1 h/day TV time change occurred on a relatively moderate level of TV viewing hours (Table 2), the impact of TV time change on waist and BMI increase may have been partly diluted. Thus, we cannot draw conclusions on the change of waist or BMI in individuals who would increase their TV time significantly more, or from a very high/low starting level. Most probably due to the same reason, as the groups were already initially on a relatively similar level, the TV time decrease did not result in a significant difference to the constantly moderate TV viewing time group.

A common limitation of most non-randomized studies is the difficulty to fully adjust for the cluster of unhealthy behaviors that have historically been seen with sedentary lifestyles. In this study population the quality and quantity of food intake and a large number other risk factors have been explored in a previous cross sectional study [16], and these analyses were also adjusted for selected other factors known to affect body weight.

Categorization of subjects according to TV time excluded 428 participants, which could potentially cause bias related to the cut offs. In the attrition analysis performed the excluded subjects were more often younger men, who watched more TV, but who had no difference in BMI or waist circumference (data not shown). According to this, the direction of the bias, if any, could only slightly dilute the results of this study. As far as the participants lost to attrition in the whole Young Finns study are concerned, they have been evaluated on several occasions and in detail after the 2001 follow-up, when the baseline characteristics between the subjects lost to follow-up and participants were compared [30]. No significant differences affecting our analyses have been seen.

The overall number of participants in this study was relatively high (1387 eligible subjects), and also the number of subjects in each subgroup remained adequate to allow more detailed analyses. Three measurements from 2001, 2007, and 2007 do not fully substitute for “continuous” assessment of TV watching and sedentary behavior, but for such a long follow-up time a more detailed TV-time analysis (variability from week to week, or from month to month) is often not possible.

Shorter bouts and breaks during longer sitting have been reported to reduce the harmful associations seen with sedentary behavior [36], [38]. The duration of individual sitting bouts or breaks during them could not be evaluated in this study.

The strength of this study are the repeated measurements and long follow-up, and the fact that groups with constantly high, moderate and low, as well as changing amounts of TV time, could be studied. A further strength of the study is the large, population-based cohort of carefully examined participants. Also the confirmation of equal BMIs in the different TV time groups 14 years prior to the 10-year follow-up add to the reliability of the results, as does the supplementing of traditional temporality criterion with the novel causality algorithms.

We found that the results from the traditional causality explorations were in line with the pairwise causality statistics, and the assumptions of pairwise causality estimation were mostly satisfied. The pairwise causality statistics were used to supplement the traditional temporality criterion, and they have been previously tested in simulated and real benchmark data sets [28], [29]. In this study, only kernel- and DirectLiNGAM-based measure provided useful information, whereas the previously recommended [29] approximate-entropy approach to asymptotic likelihood ratio did not reach statistical significance. The previous study, however, did not base its recommendation on simulations of partial confounding [29]; a situation where the kernel- and DirectLiNGAM-based approach excelled [28]. Hence, presence of partially confounding unobserved factors is a possible reason for differences between the two causality algorithms. In addition, a stronger statistical power was seen for the kernel-based causality estimator (Text S1). This may also explain why it seemed to work better in our study. When results were obtained, however, they invariably suggested TV time as a dominant causal antecedent of weight gain in the population rather than the other way around. Partial confounding effects may be of interest for future studies aiming to understand the differences between the kernel-based and entropy-approximation methods. We also considered using Patrick Hoyer's latent-variable LiNGAM method [39] to deal with latent confounding, but as the experience with it is still very limited, we recommend the method to be evaluated further prior to applying it more widely.

Both domestic and working lives are becoming less physically demanding and more sedentary [40]. However, there is no consensus on how sedentary time could be effectively reduced in our society. Controlled intervention studies can be used to evaluate the effect of reduced TV time as part of long-term weight control, but they are very difficult to conduct in practice, and can include only a limited number of participants. Short term interventions, like the one by Saunders [41], may only provide limited answers on the long-term effect of reduced sedentary time, since the counterbalancing capacity of a healthy body may prevent many adverse effects for a period of time. It also remains unclear how much reduction in sedentary time is beneficial, and through which mechanism sedentary lifestyle primarily inflicts its adverse health effects. Based on our results already a ≥1-hour decrease in TV time may have a positive impact on waist and BMI. Other measures in addition to BMI and waist circumference to further clarify the deleterious health effects of a sedentary lifestyle, and the mechanisms through which sedentary behavior impacts our health, are needed.

## Summary and Conclusions

In summary, this study provides information based on a novel exploration on causal relationship and the long-term impact of TV viewing time on waist circumference and BMI. Individuals who watch less TV gather less weight during a 10-year period. The results suggest that TV time is antecedent to larger waist and BMI, and that sedentary lifestyle is an independent risk factor increasing body weight through mechanisms that remain to be clarified. The obesogenic effect of TV viewing may be partially mediated by other behaviors and unhealthy lifestyle (16), being one of clustered bad habits. Our findings, and results from many interventional studies [20]–[22] suggest that reduction of TV time may be effective in long- or short-term weight change and weight management.

## Supporting Information

Table S1 **Relative risk of obesity associated with TV viewing time.**
(DOCX)

Text S1 **Approximate Power Calculations for Causality Estimation.**
(DOC)
