# Supplementary material for: Exploring Causality between TV Viewing and Weight Change in Young and Middle-Aged Adults. The Cardiovascular Risk in Young Finns Study
Source: PLoS One. 2014 Jul 16;9(7):e101860. doi: 10.1371/journal.pone.0101860 (PMC4100757; doi:10.1371/journal.pone.0101860)
Supplement: Table S1 — Relative risk of obesity associated with TV viewing time. (DOCX) [file pone.0101860.s001.docx]

Suppl. Table 1. Relative risk of obesity (defined as BMI >30 kg/m^2^) associated with TV viewing time. Analyses adjusted with age, sex, physical activity, energy intake, and smoking.

| **TV viewing time between 2001 and 2011 (n)** | **Relative Risk (95% CI; p-value)** |
| --- | --- |
| **Constantly low (200)** | 1.00 |
| **Constantly moderate (238)** | 2.1 (1.2-3.5; 0.007) |
| **Constantly high (84)** | 3.1 (1.7-5.7; 0.0002) |
| **Increased (221)** | 2.0 (1.2-3.4; 0.01) |
| **Decreased (216)** | 1.9 (1.1-3.4; 0.02) |

BMI = Body mass index

n = All available observations for the variable in question

Constantly low = TV time <1 h/day in 2001, 2007 and 2011

Constantly moderate = TV time >1h, but <3 h/day in 2001, 2007 and 2011

Constantly high = TV time >3 h/day in 2001, 2007 and 2011

Increased = TV time increased with >1 h/day between 2001 and 2011

Decreased = TV time decreased with >1 h/day between 2001 and 2011
