# Supplementary material for: Exploring Causality between TV Viewing and Weight Change in Young and Middle-Aged Adults. The Cardiovascular Risk in Young Finns Study
Source: PLoS One. 2014 Jul 16;9(7):e101860. doi: 10.1371/journal.pone.0101860 (PMC4100757; doi:10.1371/journal.pone.0101860)
Supplement: Text S1 — Approximate Power Calculations for Causality Estimation. (DOC) [file pone.0101860.s002.doc]

**Supplementary Text: Approximate Power Calculations for Causality Estimation**

Statistical power for causality detection can be estimated in situations that approximate ones' observed data, although the process is slow. In a previous manuscript, we studied the error rates in causality estimation by fitting statistical distributions to observed data, and by then simulating new data with known properties from these distributions that were close to the empirically observed ones (1). Here we performed a similar procedure (with code provided in the appendix below), estimating data distributions with the ones given in the below Figure S1. In order to use the code of the appendix, one needs to obtain the computing resources provided in previous works (1–3) and set them available in ones' local Matlab path.


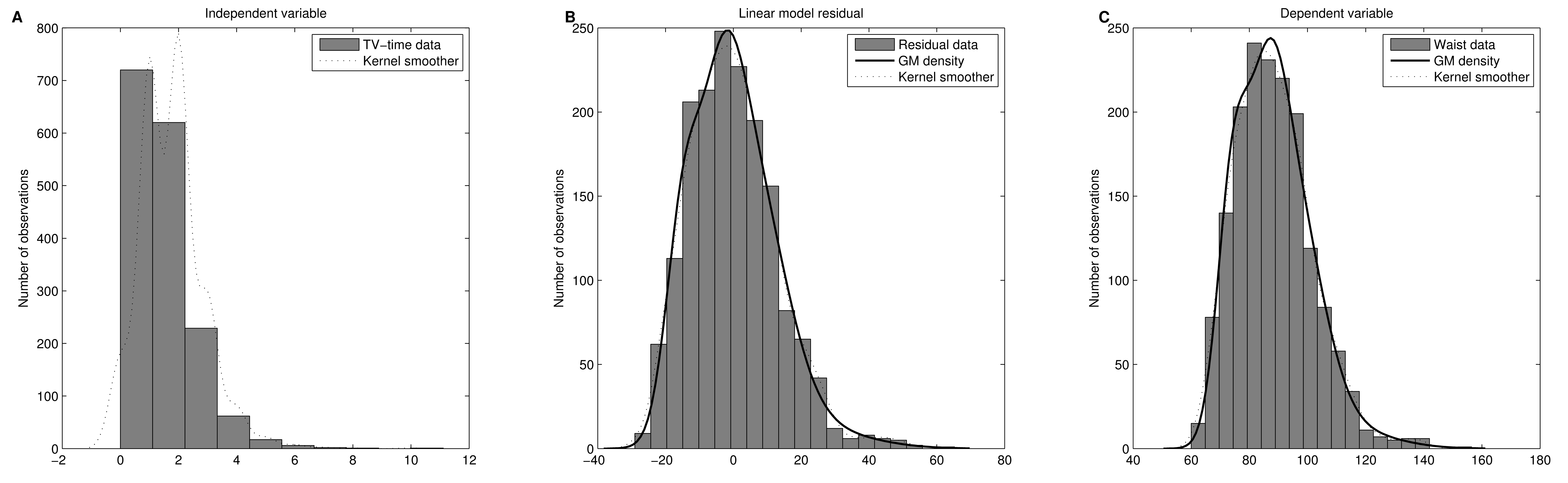


*Figure S1. Observed and Estimated Distributions. Panel* **A** *shows the distribution of TV time in 2007, and a continuous Kernel-density estimate. As the TV-time variable had only 11 different levels in that year, the TV-time distribution can be simulated from multinomial distribution with event probabilities corresponding to the empirically onserved ones (i.e., to the histogram bars). Panel* **B** *shows the distribution of the residual of the year-2007 Waist-length variable when regressed onto the TV time, its Kernel-density estimate, and a Gaussian-Mixture (GM) density of four Gaussians fitted to the observed data and approximating the observed non-Gaussian distribution. For completeness, panel* **C** *shows the same estimates for the (assumed) dependent Waist-length variable. Multinomial and Gaussian-Mixture densities shown in panels* A *and* B *can be used for creating random samples of* x *and* e*, respectively, with* y = bx + e *being the causally descendent variable and* b *the strength of the linear association between the two variables. This setup can then be used for testing the statistical power of causality algorithms for detecting the causal antecedent* x*, when it is not known* a priori*.*

Given the distribution estimates of Figure S1, we estimated the power to detect the causal direction by simulations described in the appendix. In this case, we found that the correct causal dimension was detected in 100 out of the 100 simulated data sets of the same sample size as the observed data when using 0.05 significance level and the kernel-based causality algorithm, and only in 41 of the same data sets when using the algorithm based on the entropy approximation, yielding an approximate statistical power of 1 and 0.41 for the two algorithms, respectively. The computation took 13 hours and 12 minutes on Intel i7-core and Linux version 7.10.0 (R2010a) of Matlab® software. The result pertains to an ideal situation with an unambigous (although unknown) causal direction. See previous research for discussion and techniques regarding less idealized settings (1–3).

**References**

1. Rosenström T, Jokela M, Puttonen S, Hintsanen M, Pulkki-Råback L, Viikari JS, et al. Pairwise measures of causal direction in the epidemiology of sleep problems and depression. PLoS ONE. 2012;7(11):e50841.

2. Hyvärinen A, Smith SM. Pairwise likelihood ratios for estimation of non-Gaussian structural equation models. J Mach Learn Res. 2013;14:111−152.

3. Shimizu S, Inazumi T, Sogawa Y, Hyvärinen A, Kawahara Y, Washio T, et al. DirectLiNGAM: a direct method for learning a linear non-Gaussian structural equation model. J Mach Learn Res. 2011;12:1225–48.

**Appendix – A Matlab code for variables 'tvtime' and 'waist'**

% StudyPower.m

disp('Fitting and plotting simulation distributions');

r = regstats(waist,tvtime);

options = statset('MaxIter',1000);

gmfit = gmdistribution.fit(r.r,4,'Options',options);

gmfitw = gmdistribution.fit(waist,4,'Options',options);

p=zeros(1,11); for i=1:11; p(i)=mean(tvtime==(i-1)); end; p

%%

figure;

subplot(1,3,1);

edg = linspace(min(tvtime),max(tvtime),10); [f,xi] = ksdensity(tvtime);

n = histc(tvtime,edg); bar(edg,n,'histc'); colormap gray;

set(get(gca,'Children'),'FaceColor',[0.5 0.5 0.5]); hold;

plot(xi,(edg(10)-edg(1))*length(tvtime)/9*f,'k:','LineWidth',0.5);

set(gca,'FontSize',10);

ylabel('Number of observations');

legend('TV-time data','Kernel smoother');

title('Independent variable');

annotation('textbox',[0.1, 0.87, 0.1, 0.1],'String','A',...

'FontSize',12,'LineStyle','none','FontWeight','bold');

subplot(1,3,2);

edg = linspace(min(r.r),max(r.r),20); [f,xi] = ksdensity(r.r);

n = histc(r.r,edg); bar(edg,n,'histc'); colormap gray;

set(get(gca,'Children'),'FaceColor',[0.5 0.5 0.5]); hold;

plot(xi',(edg(20)-edg(1))*length(r.r)/19*pdf(gmfit,xi'),'k','LineWidth',1.5);

plot(xi,(edg(20)-edg(1))*length(r.r)/19*f,'k:','LineWidth',0.5);

set(gca,'FontSize',10);

ylabel('Number of observations');

legend('Residual data','GM density','Kernel smoother');

title('Linear model residual');

annotation('textbox',[0.39, 0.87, 0.1, 0.1],'String','B',...

'FontSize',12,'LineStyle','none','FontWeight','bold');

subplot(1,3,3);

edg = linspace(min(waist),max(waist),20); [f,xi] = ksdensity(waist);

n = histc(waist,edg); bar(edg,n,'histc'); colormap gray;

set(get(gca,'Children'),'FaceColor',[0.5 0.5 0.5]); hold;

plot(xi',(edg(20)-edg(1))*length(waist)/19*pdf(gmfitw,xi'),'k','LineWidth',1.5);

plot(xi,(edg(20)-edg(1))*length(waist)/19*f,'k:','LineWidth',0.5);

set(gca,'FontSize',10);

ylabel('Number of observations');

legend('Waist data','GM density','Kernel smoother');

title('Dependent variable');

annotation('textbox',[0.67, 0.87, 0.1, 0.1],'String','C',...

'FontSize',12,'LineStyle','none','FontWeight','bold');

%% Check power - simulate

tic; np = 100; p_ker = zeros(np,1); p_ent = p_ker; N = length(tvtime);

for j = 1:np

n = 1000;

Tker = zeros(n,1); Tent = zeros(n,1);

s = regstats(waist,tvtime); r = s.beta(2);

e = random(gmfit,length(tvtime)); e = e - mean(e);

xo = mnrnd(1,p,N); [xo,xun] = find([xo==1]');

yo = r*xo + e;

for i = 1:n

% bootstrap

X = boot([xo,yo]); x = X(:,1); y = X(:,2);

% compute Kernel-based estimate

s = regstats(x,y); Jy = my_call_contrast([s.r';y']);

s = regstats(y,x); Jx = my_call_contrast([s.r';x']);

Tker(i) = Jy - Jx;

% compute approximate-entropy estimate

x = zscore(x); y = zscore(y);

J = pwling([x,y]',1); Tent(i) = J(1,2);

end

q = quantile(Tker,0.025);

if 0 < q; p_ker(j) = 1; end;

q = quantile(Tent,0.025);

if 0 < q; p_ent(j) = 1; end;

if mod(j,5) == 0; disp(['iteration: ',num2str(j)]); end;

end

toc

sum(p_ker)/np

sum(p_ent)/np

function X=boot(X)

N=size(X,1);

index=round(rand(N,1)*N+.5);

X=X(index,:);
